# Chromosome Instability and Oxidative Stress Markers in Patients with Ataxia Telangiectasia and Their Parents

**DOI:** 10.1155/2013/762048

**Published:** 2013-07-09

**Authors:** Luciane Bitelo Ludwig, Victor Hugo Valiati, Roberta Passos Palazzo, Laura Bannach Jardim, Darlan Pase da Rosa, Silvia Bona, Graziela Rodrigues, Norma Possa Marroni, Daniel Prá, Sharbel Weidner Maluf

**Affiliations:** ^1^Medical Genetics Service, Hospital de Clínicas de Porto Alegre, Rua Ramiro Barcelos 2350, 90035-903 Porto Alegre, RS, Brazil; ^2^Toxicology and Molecular Biology Laboratory, Universidade do Vale do Rio dos Sinos—UNISINOS, Avenida Unisinos, 950, 93.022-000 São Leopoldo, RS, Brazil; ^3^Experimental Hepatology Laboratory, Hospital de Clínicas de Porto Alegre—HCPA, Rua Ramiro Barcelos, 2350, 90035-903 Porto Alegre, RS, Brazil; ^4^Oxidative Stress Laboratory, Universidade Luterana do Brasil—ULBRA, Avenida Farroupilha 8001, 92425-900 Canoas, RS, Brazil; ^5^Graduate Course in Health Promotion, Universidade de Santa Cruz do Sul (UNISC), Avenida Independência 2293, 96815-900 Santa Cruz do Sul, RS, Brazil

## Abstract

Ataxia telangiectasia (AT) is a rare neurodegenerative disorder, inherited in an autosomal recessive manner. Total blood samples were collected from 20 patients with AT, 13 parents of patients, and 17 healthy volunteers. This study aimed at evaluating the frequency of chromosomal breaks in spontaneous cultures, induced by bleomycin and ionizing radiation, and further evaluated the rates of oxidative stress in AT patients and in their parents, compared to a control group. Three cell cultures were performed to each individual: the first culture did not receive induction to chromosomal instability, the second was exposed to bleomycin, and the last culture was exposed to ionizing radiation. To evaluate the rates of oxidative stress, the markers superoxide dismutase (SOD), catalase (CAT), and thiobarbituric acid (TBARS) were utilized. Significant differences were observed between the three kinds of culture treatments (spontaneous, bleomycin, and radiation induced) and the breaks and chromosomal aberrations in the different groups. The oxidative stress showed no significant differences between the markers. This study showed that techniques of chromosomal instability after the induction of ionizing radiation and bleomycin are efficient in the identification of syndrome patients, with the ionizing radiation being the most effective.

## 1. Introduction

Ataxia telangiectasia (AT) is a rare neurodegenerative disorder, autosomal recessive inherited [1, 2]. The carriers are apparently born normal; however, by 2-3 years old, clinical manifestations appear. The frequency of occurrence of the syndrome in the United States is approximately one in 40,000 live births [3].

Ataxic movements and ocular telangiectasia are among the pathological manifestations [4–7]. AT syndrome is caused by a mutation in the gene located on chromosome 11q22-23 encoding the protein ATM kinase [2, 8]. ATM recognizes double-stranded breaks in DNA and signals the cell-cycle checkpoints [2].

AT patients are sensitive to ionizing radiation [9, 10] and have chromosomal instability, defects in cell-cycle checkpoints [2, 11], and, therefore, increased risk of developing cardiovascular disease and cancer [6, 12]. Hypersensitivity and chromosomal instability characteristic of AT patients can be evaluated through the high frequency of chromosomal breaks and gaps, rearrangements, aneuploidy, and translocations [13–15] observed in cells spontaneous carriers [16] or in cells exposed to mutagens [13–15]. Furthermore, patients with AT exhibit high rates of chromosomal rearrangements involving autosomal chromosomes 7 and 14. Changes are often found as translocations and inversions involving these chromosomes [17].

Chronic oxidative stress is a common feature of AT [14, 18], but the involvement of ATM protein or the degree of oxidative stress is not elucidated. Bleomycin is already widely used in the evaluation of chromosomal instability in patients with clinical suspicion of AT, but is rarely used as a supplementary examination or routine for the parents of these patients. Based on this, the present work is to provide important information to incorporate new routine tests in identifying patients with AT. Early identification of these is of paramount importance for prenatal counseling, diagnosis, improvement or insertion and treatments for the various manifestations of the disease. The early diagnosis of AT would help in the prevention of cancer, where there is an increased frequency of this disease among patients and their parents.

In this sense, the present study evaluated the frequency of spontaneous, radiation-induced and bleomycin-induced chromosome breakage, as well as the level of oxidative stress in AT patients and parents of patients with the syndrome by comparing the results with the control group.

## 2. Materials and Methods

### 2.1. Patients and Blood Sampling

This study was developed at the Cytogenetics Laboratory from the Medical Genetics Service of the Hospital de Clínicas de Porto Alegre, Brazil, between August 2011 and June 2012. We evaluated the chromosomal instability and oxidative stress in blood cells sampled from 20 AT patients, aged 15.3 ± 10.2 years and 13 parents aged 41.0 ± 10.6 years of the AT patients. The control group was composed of 17 healthy individuals aged 22.4 ± 13.4 years.

Subjects were enrolled after signing an informed consent term in accordance with the guidelines of the Ethics Committee at Hospital de Clínicas de Porto Alegre (HCPA), RS, Brazil. Ten-milliliter samples of heparinized peripheral blood were collected, immediately protected from light, and stored at 4°C for evaluation. All subjects answered the personal health questionnaire of the *Commission for Protection Against Environmental Mutagens and Carcinogens* (ICPEMC). 

### 2.2. Cell Cultures, Treatments, and Chromosome Preparations

For the chromosomal instability analysis, three cell cultures were performed to each individual included in this study. A protocol adapted from 1960 [19] was used. Slides were stained with GTG-banding technique [20].

The first culture did not receive induction to chromosomal instability, thereby representing the spontaneous cultures of each individual. The second culture was exposed to bleomycin (6 *μ*g/mL of culture). The third culture was exposed to 3 Gy (ionizing radiation from a ^137^Cs *γ*-ray source). The irradiation was performed at the Gamma Cell 1000 Elite irradiator.

The breaks and chromosomal aberrations observed in the three treatments of all individuals groups were reported. A minimum of 4 and a maximum of 50 metaphases made by culture were analysed, depending on the cell growth of each culture. Chromosomal aberrations were classified as chromatid breaks (chtb), chromosome breaks (chrb), chromosome fragments, translocations (t), deletions (del), interstitial deletions, dicentric chromosomes, triradial and quadriradial figures, Rings chromosome (r), Additional materials (add), isochromosomes (iso), chromatid failures (chtg), chromosomal failures (chrg), and fragile sites. 

To calculate the frequency of chromosomal instability flaws and fragile sites were excluded, because they are not considered chromosome breakage. The other changes were included in the count, and the number of breaks for each occurrence of each chromosome abnormality observed was recorded.

### 2.3. Definition of Chromosomal Changes

The chromosome breaks are defined as all discontinuities in the chromosome that are experiencing a greater distance than the width of a chromatid, which may involve one (chtb) or both chromatids (chrb) breaks giving rise to acentric chromosome fragments [21]. The translocations are formed when there is an exchange of portions of two or more chromosomes [22]. Deletions are characterized by the loss of a portion of a chromosome. These deletions can be terminal (del) or interstitial (interstitial deletion). Interstitial deletions are defined when there is loss of a middle segment of the chromosome [22]. The chromosomes are dicentric chromosomes with two centromeres. They are formed from two chromosomes break and union portions of centromere [22]. The figures are chromosomal rearrangements involving more than one chromosome and more than one break point, which can be classified as either triradial or quadriradial [23]. The chromosomal rings are formed when there is loss of telomeric portions of chromosomes resulting from the union of edges forming a circular chromosome [22]. Additional materials of unknown origin were classified as add. These additional materials can be formed by insertion, duplication, or translocation of chromosome segments [22]. Isochromosome is formed when a loss occurs in a portion of the chromosome and the other portion is doubled [22]. Failures chromatid and chromosomal are defined as all discontinuities in one (chtg) or both chromatids (chrg) of chromosomes that have a distance less than one chromatid [21]. The fragile sites are regions where there is abnormal chromatin compaction [24]. They are viewed as a glitch in the chromosome.

### 2.4. Oxidative Stress

For preparation of the samples, 5 mL of whole blood was collected from each patient. The samples were centrifuged for 5 minutes at a speed of 3000 RPM. After centrifugation, plasma was separated from red blood cells using a disposable Pasteur pipette. The plasma was stored in an Eppendorf.

Red blood cells were centrifuged for 5 minutes at a speed of 3000 RPM and washed three times with saline solution. After washing, the red blood was collected and stored in a 1.5 mL Eppendorf with a buffer prepared with magnesium sulfate (MgSO_4_) and acetic acid. The Eppendorf's plasma and red blood cells were stored in a freezer at −80°C until processing.

To assess oxidative stress, markers TBARS (thiobarbituric acid), SOD (superoxide dismutase), and CAT (catalase) were used. 

### 2.5. Measure of Substances That React in TBARS

The technique of TBARS is the sample heating with thiobarbituric acid and the consequent formation of a colored product, measured in a spectrophotometer at 535 nm. The occurrence of staining is due to the presence of Malondialdehyde (MDA: an indicator of oxidative stress) and other substances from lipid peroxidation in biological material. Plasma samples were placed in test tubes with 0.75 mL of trichloroacetic acid (TCA) 10%, 0.25 mL of homogenate, 0.5 mL of thiobarbituric acid (TBARS) 0.67%, and 25 mL of distilled water. The tubes were stirred and heated to a temperature of 100°C. After the tubes were cooled, 1.5 mL of n-butyl alcohol was added, to extract the pigment formed. After that the samples were placed in a shaker (Biomatic) for 45 seconds and centrifuged for 10 minutes at 3000 RPM (1110 ×g). Finally, the colored product was removed and read in spectrophotometer (CARY 3E-UV-Visible Spectrophotometer Varian) with a wavelength of 535 nm. The concentration of TBARS obtained was expressed in ng per mg total protein [25, 26].

### 2.6. Evaluation of the Activity of CAT

The rate of decomposition of hydrogen peroxide (H_2_O_2_) is measured spectrophotometrically at 240 nm, and this wavelength H_2_O_2_ has maximum absorbance. An incubation mixture containing a final volume of 1000 *μ*L with reagents was prepared: 50 mM phosphate buffer, pH 7.4 and 0.3 M H_2_O_2_. In quartz cuvette, 955 *μ*L phosphate buffer and 25 *μ*L plasma were added, which were placed in the apparatus and minus the blank. 20 *μ*L of hydrogen peroxide was added and readings were performed at 240 nm. The results were expressed as pmoles/g tissue per mg total protein [26, 27].

### 2.7. Evaluation of the Activity of SOD

The technique for determination of SOD, second Misra and Fridovich [28], is based on inhibition of the formation of superoxide dismutase in adrenochrome autoxidation of epinephrine. Whereas epinephrine remains stable in acidic solutions and spontaneously oxidizes in basic solutions, favoring the formation of adrenochrome, SOD can be measured spectrophotometrically by following the change in absorbance at 480 nm of epinephrine which has a peak absorbance. To perform the reaction, a mixture was prepared with a final volume of 1 mL with bicarbonate buffer (0.05 M, pH 10.2) and plasma epinephrine (4 mM). The absorbance used was 480 nm, at 30°C. The line pattern was developed with increasing concentrations of SOD (20–100 nM) to determine the concentration that produces the same whether the inhibition of autoxidation of epinephrine by 50%, and the results were expressed as USOD/mg of total protein. An enzyme activity is defined as the amount of enzyme which is able to inhibit 50% of autoxidation of epinephrine [26].

### 2.8. Quantification of Total Protein

For the quantification of total protein, the method of Bradford [29] was used. The Bradford method is a technique for determining total protein using the Coomassie Brilliant Blue dye BG-250. This method is based on the interaction between the dye BG-250 proteins and macromolecules containing amino acids basic side chains or aromatic amino acids. In the reaction pH, the interaction between the protein and the high molecular weight dye BG-250 causes the displacement of the equilibrium to form anionic dye, which absorbs strongly at 595 nm in a spectrophotometer [30].

### 2.9. Statistical Analysis

To evaluate the difference of the frequencies of chromosomal breaks between the different treatments (Spontaneous, bleomycin, and radiation) in each group (patients, parents, or controls) the Friedman test was used. 

Statistical analysis of chromosomal abnormalities and total number of breaks between different groups and treatments were performed using the Kruskal-Wallis test. The correlation between total chromosome breakage and different oxidative stress markers was performed using the Spearman test. When statistical analysis showed significant results, post hoc Bonferroni correction was concucted. Multiple comparisons were performed with SPSS, version 15.0, with a significance level of 0.05.

## 3. Results and Discussion

The sample number and analyzed cells varied depending on the difficulty of each individual cell growth. The rates of cell growth were variable among individuals sampled and from culture to culture. Overall, we observed a sharp decline in growth in the cultures of the group of patients among the three treatments, as described next. Cell growth failure was observed: in patients: 45% samples included in spontaneous and radiation treatments, and 50% samples on treatment with Bleomycin; in parents: 7.7% samples in spontaneous and radiation treatments, and 23.08% in the sample on treatment with Bleomycin and in controls: 23.53% samples in treating spontaneous and 11.76% in the treatment with radiation and Bleomycin.

Regarding the frequency of chromosome breakage, the average of those in the spontaneous treatment of patients with AT demonstrated to be increased compared to the control group, as shown in Table 1. In relation to treatment with induction of DNA damage, patients with AT demonstrated hypersensitivity to the effects of bleomycin and ionizing radiation compared to the control group, confirming what has been described by Huo et al. [9], Sun et al. [10], Abraham [31], and Barzilai et al. [32] (Table 1). Hypersensitivity for bleomycin was demonstrated to be about three times higher than that recorded in the parent groups and controls. Already, hypersensitivity to ionizing radiation proved to be about four times higher in the group of patients than that observed in the groups of parents and controls.

The average frequency of chromosome breakage in parents was almost similar to that obtained by the control group, showing no significant differences as observed in the study by Sun et al. [10] (Table 1).

 Statistically, the frequency of chromosomal breaks observed in the three treatments showed significant differences (*α* = 0.05) in patients, parents, and controls, as shown in Table 2, and in the breaks and chromosomal aberrations in the different groups (Table 3). Increased rearrangements were not observed involving the chromosomes 7 and 14. 

Statistically, the total number of breaks in each of the different treatment groups showed significant differences (Table 4). In spontaneous treatment, differences were observed between patients and controls, and for treatment with bleomycin and radiation, such differences were observed between patients and parents and between patients and controls (Table 4).

No significant differences were observed between different markers of oxidative stress, as shown in Table 5. 

The correlation analysis between the total number of breaks of the spontaneous treatment in each group relative to each index marker of oxidative stress showed no significant differences, as shown in Table 6.

A factor that may have influenced the growth of these cultures was the amount of DNA damage generated after induction with bleomycin and radiation. The damage caused to the DNA interferes with their replication, triggering mutations, various types of chromosomal abnormalities, or cell death by apoptosis [33]. For microscopic analysis, cells are stationed in metaphase by the use of colchicine—in this stage, the cells have been through the process of repairing damage (G_2_ phase), where cells that have undergone higher levels of DNA damage are eliminated via apoptosis. Gilad et al. [34] showed that ionizing radiation inhibited cell growth in the 6 patients who were included in the study, proving the difficulty of cell growth in patients with the syndrome.

The variation in the amount of cells analyzed for each patient may have influenced the low rates of chromosomal rearrangements involving chromosomes 7 and 14 in patients with AT observed. It was then possible to observe that the evaluation techniques of chromosomal instability after exposure to ionizing radiation or bleomycin are effective in identifying patients with the syndrome, and ionizing radiation was considered the most effective. 

Prospects are expected to increase the sample size to conduct further sampling of patients and standardizing the number of cells analyzed. A much more detailed analysis of each patient was developed in order to make more accurate assessments by chromosomal abnormalities and indices of oxidative stress. The inclusion of the analysis of the marker of oxidative stress glutathione peroxidase (GPx) is also expected. The GPx develops its role in situations where there are low levels of hydrogen peroxide, while CAT operates in situations of high concentrations [35, 36]. Therefore, with the inclusion of this marker and with the addition of a larger number of patients, it will be possible to measure reliably whether or not this happening oxidative stress is strong evidence in patients with AT.

## Figures and Tables

**Table 1:** Frequency of chromosomal breaks observed in the different groups and treatments.

Frequency of chromosomal breaks			
	Spontaneous	Bleomycin (6 *μ*g/mL)	Radiation (3 Gy)
Patients			
Frequency (min × max)	0.00–1.30	0.43–5.65	0.47–7.32
*χ* frequencies/cell	0.18	1.63	2.10
*N*° cells (min × max)	30 × 50	30 × 50	30 × 50
Parents			
Frequency (min × max)	0.00–0.13	0.00–1.27	0.23–0.93
*χ* frequencies/cell	0.02	0.56	0.52
*N*° cells (min × max)	30 × 50	5 × 50	21 × 50
Control group			
Frequency (min × max)	0.00–0.03	0.00–0.90	0.10–0.93
*χ* frequencies/cell	0.01	0.47	0.56
*N*° cells (min × max)	30	4 × 30	13 × 30

*χ*: average frequency of chromosomal breaks per cell.

**Table 2:** Statistical analysis comparing the frequency of breaks of different treatments for each group.

Groups	Treatments			*P* value of the Friedman test
	Spontaneous	Bleomycin (6 *μ*g/mL)	Radiation (3 Gy)	
Patients	a	a, b	b	0.006
Parents	a	b	b	0.001
Control group	a	b	b	0.000

Treatments with at least one letter in common exhibit similar frequency of breaks (*α* = 0.05).

**Table 3:** Statistical analysis comparing the chromosomal aberrations (CAs) between the different groups within each treatment.

Treatments	Chromosomal aberrations	*P* value of the Kruskal-Wallis test
Spontaneous	Chromatid breaks (chtb)	0.910
Bleomycin (6 *μ*g/mL)		0.007*
Radiation (3 Gy)		0.000*
Spontaneous	Chromosome breaks (chrb)	0.289
Bleomycin (6 *μ*g/mL)		0.036*
Radiation (3 Gy)		0.197
Spontaneous	Chromosome fragments	0.022*
Bleomycin (6 *μ*g/mL)		0.009*
Radiation (3 Gy)		0.270
Spontaneous	Translocations (t)	0.097
Bleomycin (6 *μ*g/mL)		0.100
Radiation (3 Gy)		0.004*
Spontaneous	Deletions (del)	0.002*
Bleomycin (6 *μ*g/mL)		0.073
Radiation (3 Gy)		0.004*
Spontaneous	Interstitial deletions	1.000
Bleomycin (6 *μ*g/mL)		0.293
Radiation (3 Gy)		1.000
Spontaneous	Dicentric chromosomes	1.000
Bleomycin (6 *μ*g/mL)		0.439
Radiation (3 Gy)		1.000
Spontaneous	Triradial figures	1.000
Bleomycin (6 *μ*g/mL)		0.005*
Radiation (3 Gy)		0.001*
Spontaneous	Quadriradial figures	1.000
Bleomycin (6 *μ*g/mL)		0.228
Radiation (3 Gy)		0.222
Spontaneous	Ring chromosomes (r)	1.000
Bleomycin (6 *μ*g/mL)		0.587
Radiation (3 Gy)		0.943
Spontaneous	Additional materials (add)	1.000
Bleomycin (6 *μ*g/mL)		1.000
Radiation (3 Gy)		0.287
Spontaneous	Isochromosomes (iso)	1.00
Bleomycin (6 *μ*g/mL)		0.293
Radiation (3 Gy)		1.00
Spontaneous	Chromatid failures (chtg)	0.027*
Bleomycin (6 *μ*g/mL)		0.461
Radiation (3 Gy)		0.014*
Spontaneous	Chromosomal failures (chrg)	0.557
Bleomycin (6 *μ*g/mL)		0.021*
Radiation (3 Gy)		0.461
Spontaneous	Fragile sites	0.591
Bleomycin (6 *μ*g/mL)		0.314
Radiation (3 Gy)		0.004*

*demonstrates the chromosomal aberrations with statistically significant differences.

**Table 4:** Statistical analysis comparing the total number of breaks in each treatment between the different groups.

Treatments	Groups			*P* value of the Kruskal-Wallis test
	Patients	Parents	Control group	
Spontaneous	a	a, b	b	0.007
Bleomycin (6 *μ*g/mL)	a	b	b	0.001
Radiation (3 Gy)	a	b	b	0.001

Treatments with at least one letter in common exhibit similar number of breaks (*α* = 0.05).

**Table 5:** Average indices of oxidative stress observed.

	TBARS (ng/mg prot.)	CAT (pg/mg prot.)	SOD (USOD/mg prot.)
*χ* Patients	3.36	1.11	21.16
*χ* Parents	3.03	0.95	27.04
*χ* Control group	3.42	0.73	20.79

*χ*: average indices of oxidative stress observed.

**Table 6:** Correlation of the total number of spontaneous breaks of treatment of each group in relation to indexes of different markers of oxidative stress.

Groups	Markers of oxidative stress			
	TBARS	CAT	SOD	
Patients	0.774	0.518	0.200	*P* value of the Spearman correlation
Parents	1.000	0.809	0.499	
Control group	0.222	0.223	0.631	

(*α* = 0.05).
